# A rare coincidence of presentation of TIA like illness immediately followed by acute myocardial infarction and cardiac arrest: a case report

**DOI:** 10.1186/s12245-025-00982-5

**Published:** 2025-09-30

**Authors:** Meiling Wang, Danping Yan, Sa Wang, Yuwei Wang

**Affiliations:** 1 Nursing Department, The Second Affiliated Hospital Zhejiang University School of Medicine, Hangzhou, China; 2 Emergency Department, The Second Affiliated Hospital Zhejiang University School of Medicine, Hangzhou, China

**Keywords:** Acute myocardial infarction, Acute cerebral infarction, Transient ischemic attack, Cardiocerebral infarction, Atypical manifestations, Case report

## Abstract

A 64-year-old man presented with acute left-sided limb weakness and no abnormalities on brain CT perfusion imaging. The patient soon experienced cardiac arrest, and coronary angiography after successful cardiopulmonary resuscitation demonstrated complete occlusion of the anterior descending branch and circumflex branch mid-sections, as well as diffuse lesions in the right coronary mid-section. Atypical AMI manifestations complicate emergency diagnosis, necessitating high clinical vigilance and broad differential assessment during initial evaluation.

## Background

Acute myocardial infarction(AMI) and acute cerebral infarction(ACI) are common critical and urgent diseases of the cardiovascular and cerebrovascular system. Both share many common or similar characteristics in terms of pathogenic factors, pathogenesis and pathological basis. They may complicate each other and can occur concomitantly. The treatment time window is narrow, and timely identification, diagnosis and intervention are required in clinical practice [1]. The typical symptom of AMI is chest pain, which often radiates to the left arm, neck or jaw. Some patients may present with atypical symptoms such as headache, abdominal pain, nausea, dyspepsia, dyspnea, weakness and syncope [2], a small number of patients may also exhibit numbness or weakness in the arms [3]. And numbness and weakness in the limbs are one of the most common symptoms of ACI. Non-specific clinical manifestations present significant diagnostic challenges. In this report, we present a case of transient ischemic attack (TIA) like illness immediately followed by AMI and cardiac arrest (CA).

## Case

**Fig. 1:**
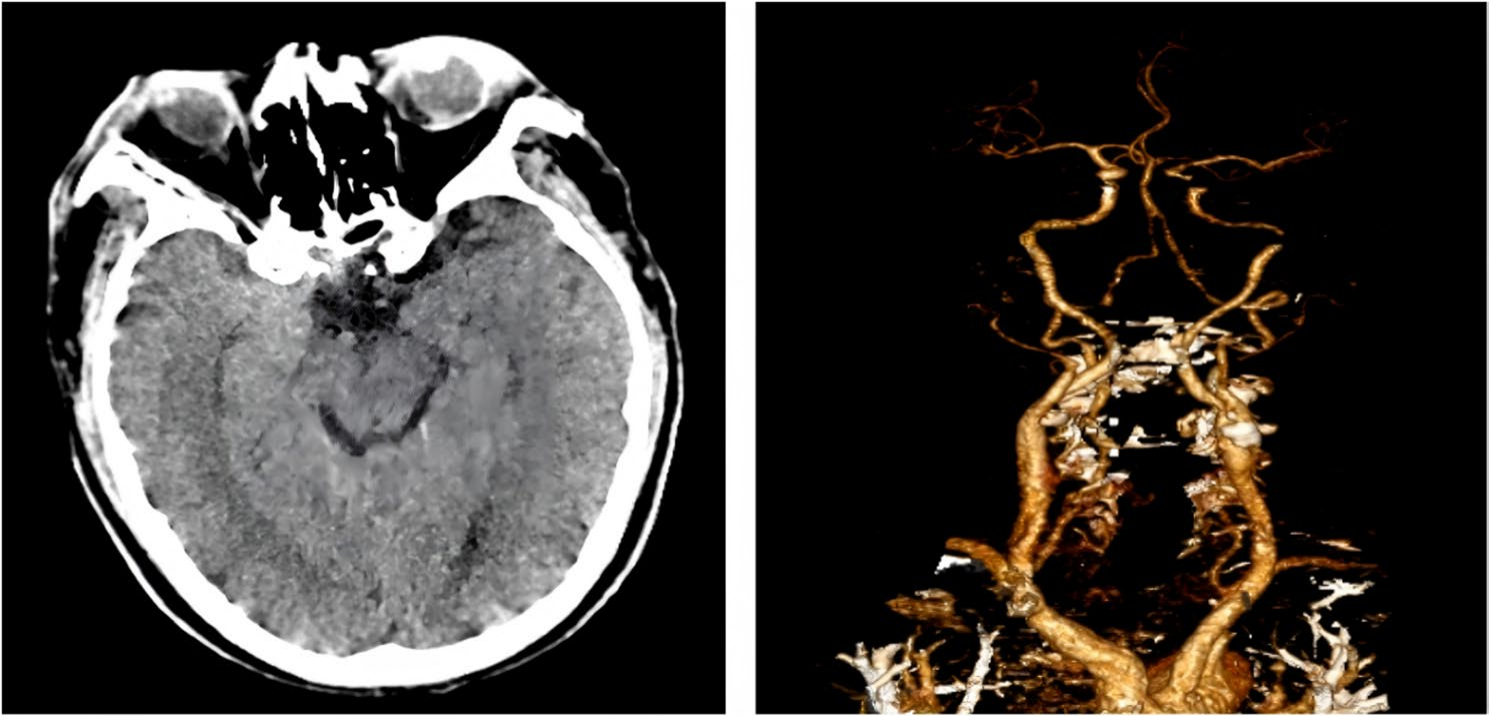
Brain CT Perfusion (CTP) Images No intracranial hemorrhage, infarction, or perfusion abnormalities

A 64-year-old male suddenly developed left-sided limb weakness 1 h before the visit. He had a history of smoking, coronary heart disease, hypertension, and a 30-year history of kidney transplantation with irregular medication use. At the time of the presentation (11:27), he was conscious, with a temperature of 36.7℃, a heart rate of 86 beats per minute, a blood pressure of 115/73 mmHg, an oxygen saturation of 86% on air, and no reports of any chest discomfort or dyspnea. Muscle strength was grade 3 in the left upper limb and grade 1 in the left lower limb, with mild numbness. His National Institute of Health Stroke Scale (NIHSS) score was 11. We immediately initiated the in-hospital stroke screening process, including the immediate establishment of intravenous access, blood laboratory tests and CT perfusion imaging of the brain (11:35). Laboratory findings on admission: white blood cell count 22*10^9^/L, hemoglobin 87 g/L, platelet count 299*10^9^/L, C-reactive protein 28.2 mg/L, creatinine 157 µmol/L, aspartate aminotransferase 98 U/L, creatine kinase 745 U/L, creatine kinase-MB 126 U/L, lactate dehydrogenase 431 U/L, troponin-T 2.12 ng/mL, glucose 14.36 mmol/L, potassium 3.63 mmol/L, sodium 141.1 mmol/L, chloride 111.2 mmol/L, calcium 2.3 mmol/L, D-dimer 2350 µg/L, prothrombin time 13.4 s, plasma fibrinogen 4.66 g/L, activated partial thromboplastin time 27.6 s. And no obvious abnormalities were found in his cerebral perfusion imaging (Fig. 1).

**Fig. 2:**
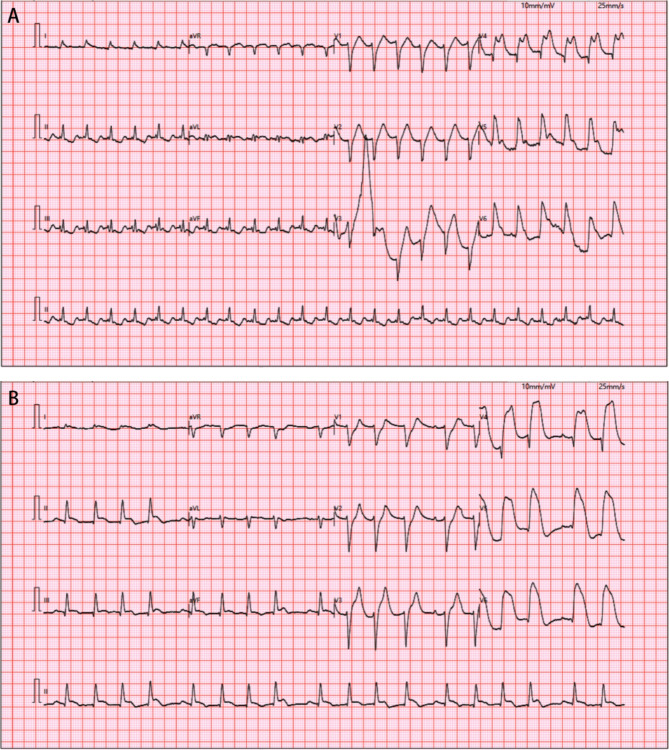
Post-ROSC Electrocardiograms. **A** First ROSC (12:31) — Sinus tachycardia with anterior ST-elevation. **B** Second ROSC (13:13) — Atrial flutter with intermittent sinus beats, second-degree AV block (Type I), poor R-wave progression, intraventricular conduction delay, and anteroinferior ST-elevation

Shortly after returning to the emergency room post-CTP, the patient experienced cardiac arrest (12:13). After 15 min of cardiopulmonary resuscitation (CPR), the patient’s spontaneous circulation was restored (12:28). We performed a bedside ultrasound (POCUS) evaluation, and the patient had no tension pneumothorax, pericardial tamponade, obvious pulmonary embolism, or aortic dissection. 28 min later, the patient experienced cardiac arrest again (12:56). After 9 min of cardiopulmonary resuscitation, the patient’s spontaneous heart rate was restored (13:05). The two electrocardiogram reports (12:31, 13:13) after return of spontaneous circulation (ROSC) showed widespread anterior lateral wall ST-segment elevation (Fig. 2). The patient exhibited hemodynamic instability, with arterial systolic blood pressure currently ranging from 70 to 80 mmHg despite norepinephrine bitartrate infusion at 133.3 µg/min.

**Fig. 3:**
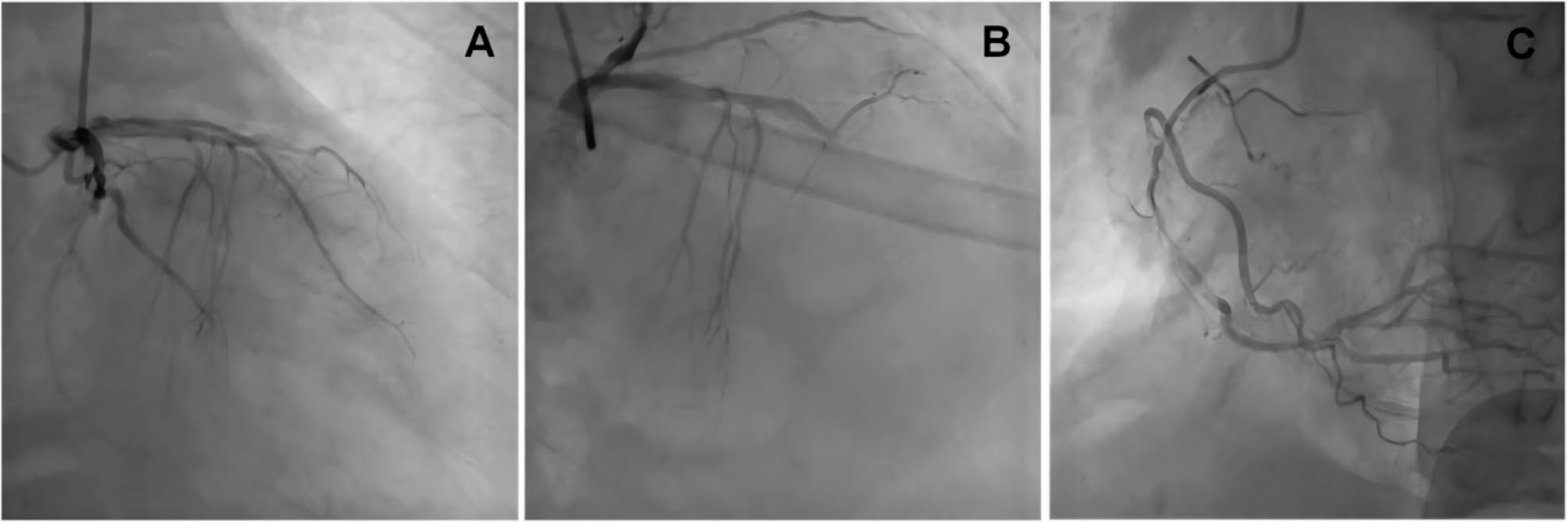
Coronary Angiographic Findings. **A** Mid-LCx chronic total occlusion (TIMI 0). **B** Mid-LAD acute total occlusion (TIMI 0). **C **Mid-RCA diffuse 95% stenosis (TIMI 3)

**Fig. 4:**
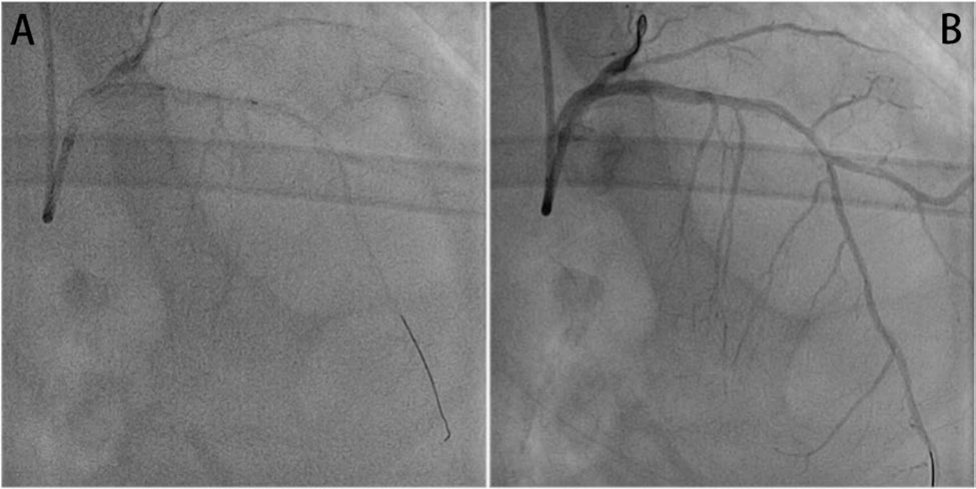
Percutaneous Coronary Intervention of Mid-LAD. **A** Pre-procedure occlusion. **B** Post-drug-coated balloon angioplasty

After loading treatment with ticagrelor 180 mg and aspirin enteric-coated tablets 300 mg, and symptomatic and supportive treatment, we initiated the cardiac catheterization room (14:09). During the percutaneous coronary intervention (PCI), the following angiographic findings were observed (Fig. 3): Acute total occlusion in the mid-segment of the left anterior descending artery (LAD), Chronic total occlusion in the mid-segment of the left circumflex artery (LCx) (both with TIMI flow grade 0). Diffuse 95% stenosis in the mid-segment of the right coronary artery (RCA) (TIMI flow grade 3). Subsequently, we performed drug-coated balloon (DCB) angioplasty on the mid-LAD lesion (Fig. 4) and initiated intra-aortic balloon pump (IABP) support. During PCI (14:41), the patient had an episode of ventricular fibrillation, which was treated with 150 J of extracorporeal defibrillation and amiodarone. The patient was transferred to the emergency intensive care unit, but the patient was hemodynamically unstable, secondary to multiple organ failure, and the family refusing medication and any resuscitative treatment. The patient died the next day.

## Discussion

We report a case of AMI complicated by cardiac arrest following left-sided limb numbness and weakness. The clinical definition of myocardial infarction (MI) denotes the presence of acute myocardial injury detected by abnormal cardiac biomarkers in the setting of evidence of acute myocardial ischemia [4]. The patients with chest discomfort or other ischemic symptoms, who develop new ST-segment elevations in 2 contiguous leads or new bundle branch blocks with ischemic repolarization patterns as an ST-elevation MI (STEMI), and patients without ST-segment elevation at presentation are usually designated non–ST-elevation MI (NSTEMI); In addition, MI can be classified into five types based on different mechanisms of occurrence [4, 5]. In this case, AMI was definitively diagnosed. The patient had a history of coronary artery disease (CAD). Initially, he developed unilateral limb numbness and weakness accompanied by a marked elevation in cardiac troponin T (cTnT), followed by cardiac arrest. The results of PCI confirmed an acute total occlusion in the mid-segment of the LAD. As the patient was assessed as a suspected stroke case without chest pain/discomfort, an initial ECG was not obtained. Despite lacking the initial admission ECG, we inferred that the patient likely experienced a Type 1 MI—defined as MI caused by atherothrombotic coronary artery disease (CAD) and usually precipitated by atherosclerotic plaque disruption (rupture or erosion). We cannot determine whether the patient had STEMI. Myocardial injury and dysfunction following cardiac arrest may result from ischemia-reperfusion injury and CPR. These processes can predispose to ventricular premature beats, ventricular tachycardia, and episodes of fibrillation, especially in the first 5 to 20 min after ROSC [6]. Therefore, ST-elevation is common post-ROSC, but ST-elevation ACS (STE-ACS) has high false-positive rates in this setting [7].

Ischemic numbness and muscle weakness in a single limb may be caused by various diseases. Progressive symptoms may suggest tumors, degenerative diseases, etc., while acute attacks are often seen in stroke [8], brain CT/perfusion imaging can rapidly exclude it. However, In this case, CT cerebral perfusion imaging of the patient did not show overt cerebral hemorrhage or infarction, but the patient had stroke-like or TIA symptoms. TIA is a high-risk warning sign for AIS. Although very rare, AMI and AIS/TIA can occur simultaneously, cardio-cerebral infraction(CCI) [9]. Studies have shown that approximately 1 out of every 200 cases of AMI and/or AIS is synchronous CCI, and the all-cause mortality rate of CCI patients is significantly higher than that of only AMI or AIS patients [10]. AMI and AIS have similar pathologic bases and co-morbidities, which can be mutually causative. High age, hypertension, diabetes, renal insufficiency, etc. are common risk factors for both [11].

The limb weakness of AMI patients may be related to hypoperfusion-induced cerebral ischemia and cardiogenic cerebral embolism [12]. Large-area myocardial infarction or severe arrhythmias such as ventricular fibrillation may lead to a sudden decline in cardiac pumping function, secondary heart failure and cardiogenic shock. In a hypotensive state, cerebral blood flow sharply decreases, and the narrowed cerebral arteries may become insufficiently perfused and occluded, potentially causing neurological symptoms including ischemic numbness and weakness [13]. However, the symptoms caused by this situation are usually diffuse or bilateral, often presenting symptoms such as dizziness and confusion. When there is severe unilateral vascular lesion, unilateral symptoms may occur, while unilateral limb symptoms caused by embolism are more common and typical. Both AIS/TIA and AMI can result from atherosclerotic plaque rupture or detachment generating emboli, causing either in situ arterial occlusion or distal artery embolism. Furthermore, intravascular stasis, inflammatory changes and hypercoagulability due to local dyskinesia of the left ventricle in infarction predispose to the formation of adnexal thrombi, emboli to the intracranial arteries may originate from cardiac sources, with atrial fibrillation (AF) being the primary cause [14]. If a thrombus detaches and embolizes the contralateral motor-sensory brain region before or very early after the occurrence of chest pain and other MI symptoms, then ischemic numbness and weakness in the single limb may become the first symptom that the patient notices [15]. On ECG Tracing 2B (Fig. 2B), we detected suspicious atrial fibrillation waveforms. Despite various interfering factors, we still hypothesized that the patient might have experienced a transient cardiogenic cerebral embolism.

AMI can induce the occurrence of AIS, and neurological events can also lead to myocardial injury and dysfunction, causing arrhythmias, stress-induced cardiomyopathy, AMI and paroxysmal hypertension, etc. as serious cardiac adverse events in acute cerebrovascular events. Abnormal ECG and elevated cardiac biomarkers are very common in AIS [16, 17]. If the symptoms of AIS and AMI are typical, their diagnosis is usually easy and clear. However, in clinical practice, there are often cases where the symptoms of one disease mask those of another. For example, in AMI patients, due to severe chest pain, they may overlook the sensory/motor abnormalities caused by AIS; in AIS patients, due to aphasia, consciousness disorders, they may fail to report their symptoms and thus miss the diagnosis of AMI [18]. When the symptoms of AIS and AMI occur simultaneously, more caution should be exercised regarding the occurrence of aortic dissection. Depending on the extent of the tear and the false lumen, the blood supply may be obstructed. When the dissection extends to the subclavian artery or iliac artery, the perfusion of the arms and legs may differ; when the dissection detachment extends to the coronary artery ostium, carotid artery, vertebral artery or basilar artery, the blood flow to the myocardium and brain is inhibited, resulting in STEMI and TIA/AIS on the electrocardiogram [19, 20]. Therefore, early differential diagnosis is extremely important, which directly affects the subsequent treatment decisions.

Diagnosis and treatment are frequently delayed in MI with atypical presentations. In this case, the suspected diagnosis of acute stroke was reasonable, we made the diagnosis through cranial CTP. However, in this situation, the recognition of the patient’s ECG was delayed. In the chaotic environment of a busy emergency department, for time-limited diseases such as AIS/AMI, the rapid assessment and accurate judgment of medical history and symptoms/signs are challenging. This is particularly true for patients with subtle or atypical symptoms, where the risk of missed or delayed diagnoses is substantially heightened. For high-risk patients with cardiovascular and cerebrovascular diseases such as the elderly, multiple comorbidities, and complex medication use, in addition to comprehensive questioning and detailed physical examination, dynamic monitoring of related indicators such as electrocardiogram and myocardial enzyme spectrum should also be conducted. Early completion of multimodal assessment and careful differentiation should be carried out to gain time for rescue.

## Conclusion

This case illustrates a rare presentation of TIA-like symptoms immediately preceding AMI and cardiac arrest. The clinical identification and diagnosis of atypical symptoms of AMI are challenging. Emergency physicians must remain highly vigilant at all times, carefully review the medical history, make differential diagnoses, and avoid missed or misdiagnosed cases. Regardless of whether there is obvious chest pain, high-risk patients with suspected acute stroke or AMI should undergo electrocardiogram, neurological examination and imaging tests as soon as possible.

## Data Availability

No datasets were generated or analysed during the current study.
